# Danggwijagyaksan for climacteric syndrome in peri- and postmenopausal women with a blood-deficiency dominant pattern: study protocol for a randomized, double-blind, placebo-controlled pilot trial

**DOI:** 10.1186/s13063-018-2443-8

**Published:** 2018-01-15

**Authors:** Mikyung Kim, Ae-Ran Kim, Hyo-Ju Park, Ojin Kwon, Joo-Hee Kim, Eun-Ji Park, Seon-Eun Baek, Jeong-Eun Yoo, Jun-Hwan Lee

**Affiliations:** 1 Clinical Research Division, Korea Institute of Oriental Medicine, 1672 Yuseongdae-ro, Yuseong-gu, Daejeon, Republic of Korea; 2 College of Korean Medicine, Sangji University, Wonju, Republic of Korea; 3 Department of Obstetrics and Gynecology, College of Korean Medicine, Daejeon University, 62 Daehak-ro, Dong-gu, Daejeon, 300-716 Republic of Korea; 4 Department of Obstetrics and Gynecology, Dunsan Korean Medicine Hospital of Daejeon University, 75 Daedeok-daero 176 beon-gil, Seo-gu, Daejeon, Republic of Korea; 5 Korean Medicine Life Science, University of Science & Technology, Campus of Korea Institute of Oriental Medicine, 1672 Yuseongdae-ro Yuseong-gu, Daejeon, Republic of Korea

**Keywords:** Danggwijagyaksan, Dangguishaoyaosan, Tokishakuyakusan, Climacteric syndrome, Menopause, Randomized controlled trial, Traditional medicine, Herbal medicine, Pattern identification

## Abstract

**Background:**

This study aims to explore the safety, efficacy, and feasibility of Danggwijagyaksan (DJS) for alleviating climacteric syndrome in peri- and postmenopausal women with a blood-deficiency dominant pattern.

**Methods/design:**

This is a randomized, double-blind, placebo-controlled pilot clinical trial. A total of 34 women with climacteric syndrome who have signed informed consent forms will be registered in this study. Placebo or DJS will be randomly assigned to the participants in an equal proportion. The participants will visit the clinical trial center every 2 weeks and receive placebo or DJS granules. The treatment period is 4 weeks and the administration frequency is three times daily. Data will be collected from the participants at baseline, at week 5, and at week 9 after random allocation. The primary outcome measure will be the mean change in the Menopause Rating Scale from baseline to week 5. Secondary outcome measures will include the World Health Organization Quality of Life-BREF (WHOQOL-BREF) score, the Blood Deficiency Scoring System score, lean body mass, and blood tests, including serum follicle-stimulating hormone and estradiol concentration. To assess the safety of DJS, a laboratory test will be conducted before and after treatment and the participants will be asked about any occurrence of adverse events every visit. The recruitment rate, completion rate, and medication adherence will also be calculated, to assess feasibility.

**Discussion:**

The findings of this study will provide the basis for a full-scale randomized controlled trial to confirm the safety and efficacy of DJS for the treatment of climacteric syndrome in peri- and postmenopausal women.

**Trial registration:**

Clinical Research Information Service (CRIS), Republic of Korea, KCT0002387. Registered on 25 July 2017.

**Electronic supplementary material:**

The online version of this article (doi:10.1186/s13063-018-2443-8) contains supplementary material, which is available to authorized users.

## Background

As the population ages and the average life expectancy of women increases, the proportion of postmenopausal women in the population also increases. Many peri- and postmenopausal women experience various symptoms, including vasomotor symptoms, such as hot flashes, sweating, or palpitations, owing to decreased ovarian function and female hormone imbalance, which is referred to as climacteric syndrome [1]. Climacteric syndrome usually begins at the perimenopause period and persists for years, maybe even 20 to 30 years after menopause [1].

According to a survey that targeted postmenopausal women in five Asian countries, more than 90% of the respondents reported that they experienced climacteric syndrome [2]. Another survey of natural menopausal women in South Korea reported similar results [3]. Climacteric syndrome is not only a very common condition among peri- and postmenopausal women; it also interferes with their activities of daily living and deteriorates their quality of life [4, 5].

Hormone therapy has been known to be the first option for the management of a variety of symptoms of climacteric syndrome, particularly vasomotor symptoms, but the range of use is limited [6]. It is contraindicated in women with active liver disease, a history of breast or endometrial cancer, unexplained vaginal bleeding, coronary heart disease, or a history of venous thromboembolic events or stroke [6]. For this reason, there is a growing demand for safe and effective nonhormonal therapies [7], and complementary and alternative therapies, such as yoga, acupuncture, and herbal medicine, are becoming increasingly popular among postmenopausal women [8].

Traditional medicine is still widely used in East Asian countries, such as Korea, China, and Japan. Doctors of traditional East Asian medicine usually use a system called ‘pattern identification’ for diagnoses and to determine how to treat a disease, based on the pattern of the symptoms and the signs observed in the patient. Danggwijagyaksan (DJS) and Gyejibongnyeonghwan are commonly prescribed herbal medicines for the management of climacteric syndrome in women in the peri- and postmenopausal periods, according to this system [9–12]. The authors are already conducting a pilot trial to explore the feasibility and the effects of Gyejibongnyeonghwan for patients with a blood stasis pattern [12]; this protocol explores the other popular herbal medicine, DJS. In traditional East Asian medicine, DJS has mainly been prescribed to patients with a dominant pattern of blood deficiency according to the system of pattern identification [9, 11]. The indications for DJS, as approved by the regulatory authorities of Korea or Japan, also include climacteric symptoms of weakness, fatigue, or easy exhaustion [10].

A clinical study conducted in Korea has shown that administration of DJS for 30 days can improve the Menopause Rating Scale scores of postmenopausal women with a blood-deficiency dominant pattern [11]. In Japan, a retrospective review of medical records suggested that sleep disturbances among peri- and postmenopausal women with deficiency dominant patterns significantly improved after 5 months of DJS treatment [9]. They also reported a minor but significant increase in lean body mass and decreased diastolic blood pressure in the subjects [9]. Another study demonstrated that DJS could effectively control headache and concomitant depressive symptoms in menopausal women [10].

Tanaka [13] demonstrated that DJS was effective in alleviating hot flashes induced by hormone therapy in climacteric women. According to results from experimental studies [14–16], this effect of DJS against climacteric syndrome seems to be due to the female hormone regulation of DJS. However, it has also been observed that DJS improved lutein deficiency in ovarian-deficient women, and that it did not cause hormonal disturbances in nonmenopausal women [15].

In addition to anticlimacteric activity, DJS has been reported to be effective for the treatment of primary dysmenorrhea [17] and anemia [18]. Recently, it has been actively studied for potential use as a promising cognitive enhancer in Parkinson’s disease [19] or Alzheimer’s disease [20] based on its neuroprotective effect in the central nervous system [19–21].

Danggwijagyaksan appears to be very useful for management of the symptoms of climacteric syndrome, but evidence based on rigorously designed randomized controlled trials is still lacking. The North American Menopausal Association has mentioned that herbal medicines such as DJS are widely used around the world as a means of nonhormonal therapy for the management of climacteric syndrome, but the lack of a scientific and clinical basis for this therapy remains [7].

The goal of this pilot study is to explore the efficacy, safety, and clinical trial feasibility of DJS for climacteric syndrome in peri- and postmenopausal women with a blood-deficiency dominant pattern.

## Methods/design

### Study design

**Fig. 1:**
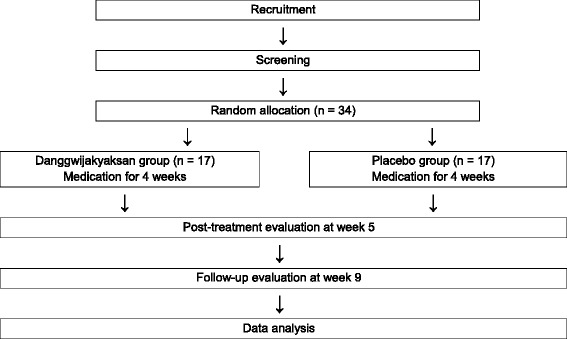
Flow chart of the study

**Fig. 2:**
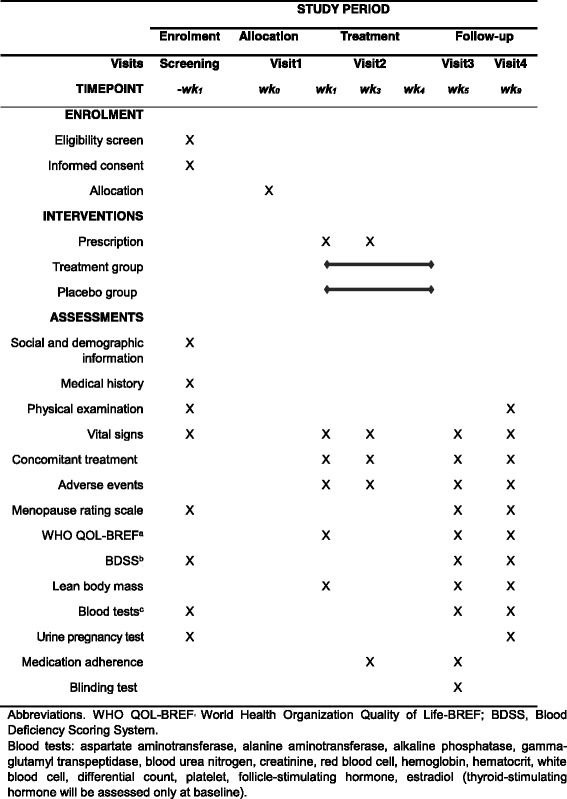
SPIRIT figure

This is a randomized, parallel-group, double-blind, placebo-controlled pilot clinical trial. Placebo or DJS will be randomly assigned, with a 50% probability, to the subjects who have provided written consent and passed the screening criteria for this study. The treatment period will be 4 weeks. Post-treatment evaluations will be performed at week 5, and then follow-up evaluations will be conducted at week 9. The study schedule is summarized in Figs. 1 and 2.

### Recruitment

This study will be conducted in the outpatient department of an obstetrics and gynecology clinic at a clinical trial center of a Korean traditional medicine hospital. To recruit target subjects, advertisements will be distributed using flyers, local newspapers, subway posters, and online and offline bulletin boards of the hospital.

### Screening

The researcher will screen the patients who have voluntarily signed the written consent form. Subjects who meet the following eligibility criteria will be accepted as participants for this study.

### Participants

#### Inclusion criteria

1. 45–60 years old
2. Presence of peri- or postmenopausal climacteric syndrome (includes natural or induced menopause)
3. A Menopause Rating Scale score of 9 or higher
4. A Blood Deficiency Scoring System score of 30 or higher
5. Willingness to participate in the clinical trial and provide written consent

#### Exclusion criteria

1. Having undergone postmenopausal hormonal therapy within 3 months of the screening visit
2. Having received traditional Korean medicine to improve symptoms of climacteric syndrome within 4 weeks of the screening visit
3. Having taken any medication or functional food to mitigate symptoms of climacteric syndrome within 4 weeks of the screening visit
4. A diagnosis of serious mental disease, such as depression or anxiety disorders, or currently taking psychotropic drugs
5. Uncontrolled thyroid disease
6. Aspartate aminotransferase, alanine aminotransferase, alkaline phosphatase, or γ-glutamyl transpeptidase levels > 1.5 times the upper normal value
7. Blood urea nitrogen or creatinine level > 1.5 times the upper normal value
8. Currently undergoing transfusion or erythropoietin therapy for severe anemia
9. Vaginal bleeding of an unknown cause after menopause
10. Lactose intolerance
11. Currently participating in other clinical trials
12. Other cases considered inappropriate for this trial by the investigator

### Randomization and allocation concealment

A statistician will generate a random allocation list using Strategic Applications Software (SAS®) Version 9.4 (SAS institute. Inc., Cary, NC, USA). The list will assign 34 subjects to one of two groups, coded A or B. The list with the random allocation codes will be sealed in an opaque envelope and stored in a double-locked cabinet.

### Blinding

A person responsible for blinding will assign treatment or placebo to the randomization codes and will then send the list with the group information to the pharmaceutical company. The pharmaceutical company will prepare the drugs for the DJS and placebo groups according to the sequence on the list. The DJS and placebo drugs will look the same and will be packed in identical form. All participants and researchers, including the coordinators, monitors, and statisticians, except for the person in charge of blinding, will be blinded to the group assignments. To verify whether participant blinding has been successfully achieved, a blinding test [22] will be performed. The group allocation will not be disclosed unless the medical attendant judges that knowledge of the medication being taken by a patient is essentially required for treatment.

### Interventions

All participants in both groups will take 3 g of medication in granular form, three times daily, during the 4-week treatment period. The participants will visit the clinical trial center every 2 weeks and receive 2 weeks’ worth of medicine each time from the investigators. The treatment group will be prescribed DJS, and the control group will receive the placebo. The placebo and DJS will be manufactured by Hanpoong Pharmaceutical (Wanju, Republic of Korea) according to good manufacturing practice standards. The pharmaceutical company will not play any direct role in this study, apart from the drug manufacturing and delivery to the clinical trial center.

Danggwijagyaksan will be prepared in the form of granules with a light brownish color. The medication will be 3 g, containing 1.7 g of DJS soft extract with 0.56 g of lactose hydrate and 1.32 g of corn starch as the excipient. The DJS soft extract will be made of the water extract of a mixture of several herbal medicines, as follows: *Angelica gigas* Nakai (1 g), *Cnidium officinale* Makino (1 g), *Paeonia lactiflora* Pallas (2 g), *Poria cocos* Wolf (1.33 g), *Atractylodes japonica* Koidzumi (1.33 g), and *Alisma orientale* Juzepzuk (1.67 g). The placebo will consist of corn starch, lactose hydrate, citric acid, ginseng incense powder, and caramel color. The placebo and DJS will be identical in appearance. Both will be light brown granules packaged in silver, opaque wrappers.

### Medication compliance monitoring

To confirm adherence to the medication, the dose that each participant actually takes will be counted. Therefore, the patients will be asked to return the empty wrapping paper of the medication and any remaining medicine at visits 2 and 3.

### Prohibition and permission for concomitant treatment

1. The use of postmenopausal hormone therapy such as estrogen, estrogen and progesterone, or tibolone for climacteric syndrome will be prohibited during the study.
2. The use of any traditional medicine intended to treat climacteric syndrome, except for the intervention of this trial, will be prohibited during the study.
3. The use of functional foods or other medications aimed at improving the symptoms of climacteric syndrome will be prohibited during the study.
4. Medications that have been continuously used for the treatment of other chronic diseases before the start of this clinical trial are allowed.
5. Physical exercise that has been routinely performed before the start of this clinical trial is allowed.
6. Any change in concomitant medications, nonpharmacological therapy, or physical exercise during the study will be recorded in a clinical report form.

### Outcome measures

#### Menopause rating scale

The Menopause Rating Scale will be assessed at baseline, week 5, and week 9. The mean change of the Menopause Rating Scale score between baseline and week 5 will be the primary outcome measure of this study.

The Menopause Rating Scale is a self-administered questionnaire developed to measure the severity of menopausal symptoms and their impact on health-related quality of life in aging women [23]. The questionnaire consists of 11 questions in three dimensions including psychological, somato-vegetative, and urogenital aspects [23]. Each item uses a 0–4-point Likert scale. A higher score corresponds to more severe symptoms [23]. Climacteric syndrome is categorized as being mild (5–8), moderate (9–16), or severe (17 and more), according to the total score [24]. The reliability and validity of the Menopause Rating Scale has been confirmed [25] and its Korean version, which is provided by the online Menopause Rating Scale network, will be used in this study [23].

#### World health organization quality of life-BREF (WHOQOL-BREF)

The WHOQOL-BREF will be used at baseline, week 5, and week 9. It is a questionnaire developed to assess the quality of life [26]. It consists of 26 items regarding physical health, psychological health, social relationships, environment, overall quality of life, and general health perceptions [26]. The reliability and validity of the WHOQOL-BREF has been confirmed [27] and a verified Korean version of the WHOQOL-BREF will be used in this study [28].

#### Blood deficiency scoring system

The Blood Deficiency Scoring System will be used to assess blood deficiency at baseline, week 5, and week 9. It is a checklist covering 12 items, such as loss of concentration, insomnia, eye fatigue, and dizziness [29, 30]. These items are the main symptoms and signs of a blood-deficiency pattern according to the theory of the pattern identification system in traditional East Asian medicine. The total scores will be calculated based on the weighted score assigned to each item. A total score of more than 30 out of 100 points is considered to demonstrate blood deficiency [29, 30]. Although the validity and reliability of this questionnaire has not yet been verified, there is no other option but to use the Blood Deficiency Scoring System to identify the blood-deficiency pattern at present [30]. In this study, a Korean-translated version of the Blood Deficiency Scoring System will be used [31].

#### Hormone test

For an objective understanding of the menopausal situation, the concentrations of follicle-stimulating hormone and estradiol in blood samples will be measured. The hormone test will be conducted at baseline, week 5, and week 9. The blood specimen will be sent to the clinical pathology department for analysis.

#### Lean body mass

Lean body mass will be measured at baseline, week 5, and week 9. A previous study [27] showed that increased lean body mass is beneficial to bone nutrition in postmenopausal women with normal levels of female hormones [32]. Terauchi et al. [9] reported a significant increase in the lean body masses of female patients with climacteric syndrome after taking DJS. In this study, lean body mass will be calculated by subtracting body fat mass (kg) from body weight (kg) as measured by a bioelectrical impedance analysis method.

#### Feasibility assessment

The recruitment rate, completion rate, and medication adherence will be assessed in this trial. The recruitment rate will be presented as a percentage of the total number of registered participants out of the total number of screened subjects. The completion rate will be presented as a percentage of the total number of subjects who completed the study out of the total number of registered participants. The medication adherence will be presented as a percentage of the target dose that was actually taken by the subjects.

#### Safety assessment

To evaluate the safety of DJS, the participants will be asked about the occurrence of any adverse events and their vital signs will be examined every visit. In addition, blood samples will be collected at baseline, week 5 and week 9 for laboratory tests, including aspartate aminotransferase, alanine aminotransferase, alkaline phosphatase, γ-glutamyl transpeptidase, blood urea nitrogen, creatinine, red blood cells, hemoglobin, hematocrit, white blood cells, differential count, and platelets. The blood specimen will be sent to the clinical pathology department for analysis.

If any adverse event is detected, the investigator will evaluate the causal relationship between the adverse event and the intervention of the study. Every adverse event will be recorded in the clinical report form with the result of the causal relationship assessment. If a serious adverse event occurs during the trial, it will be reported to the institutional review board. The institutional review board will then review the serious adverse event report, and decide whether to continue or stop the study.

### Participant withdrawal criteria

1. The subject or the legal representative of the subject withdraws the study participation agreement.
2. A violation of the eligibility criteria is detected during the study period.
3. The occurrence of a serious adverse event makes it difficult to continue the study.
4. A violation of the protocol by a subject.
5. Failure to visit the clinical trial center and receive phone calls during the planned schedule.
6. Taking medications that may affect the result of the trial without the instruction of the investigator during the study period.
7. Inappropriate progress of the study as judged by the investigator.
8. In addition to these cases, when no further medication is required because the symptoms related to climacteric syndrome have disappeared completely or enough to make daily life comfortable, the investigator can decide on early termination of the trial for a subject. In this case, after acquiring the final assessment data, the subject’s participation in the clinical study will be terminated early.

### Sample size

In a previous study [11], the mean change in the Menopause Rating Scale score was 10.8 after 30 days of DJS administration. Based on a preliminary study similar to ours [12], we assumed that the Menopause Rating Scale score of the placebo-treated group will change by 2.16, which is 20% of the mean difference of DJS group. Therefore, the mean difference between the Menopause Rating Scale scores of the DJS group and the placebo group is calculated to be 8.64. The standard deviation is estimated to be 8.012 according to the data acquired from the DJS-treatment group in the previous study [11]. Under this assumption, the required sample size was calculated to be 14 per group, with a significance level of 0.05 and a power of 0.8. Considering a dropout rate of 15%, 17 people are ultimately required per group.

### Data collection

The investigators at the clinical trial center will obtain written informed consent after explaining the outline of the trial to the potential participants and then screen the eligibility of the participants. For the eligible subjects, baseline characteristics regarding the outcome measures of this study, as well as the social and demographic information and medical background, will then be collected. After randomized group allocation and medication, outcome measures regarding the effectiveness and safety of the intervention will be obtained at weeks 3, 5, and 9. The data will be collected through the patient-reported questionnaires and the participants will be blinded to the group allocation. The results of the questionnaires completed by the subject will be transferred to the clinical report form by the researchers, who are also blinded to the group assignment.

### Statistical analysis

The statistical analysis will be performed by an independent statistician using SAS® version 9.4. When the demographic and sociological data of the subjects are continuous, they will be presented in the form of means and standard deviations. Differences between the two groups will be analyzed using Student’s *t* test (or the Wilcoxon rank sum test). Categorical data will be presented in frequencies and percentages and analyzed using the chi-square test (or Fisher’s exact test). The missing values will be processed using the multiple imputation method.

The full analysis set will be the main analysis set, and the per-protocol set will be supplementary. The full analysis set will be used to achieve the ideal intention-to-treat principle as completely as possible. It will include all randomized participants unless the following criteria are met: the eligibility criteria are not met, the test drug has not been taken, or data cannot be collected because the participant was never evaluated after random assignment. The per-protocol set will include the participants who have completed the study without any major protocol violations.

The analysis of covariance will be performed to analyze the between-groups differences of the primary and secondary outcome measures. The dependent variables will be the values acquired after intervention, the covariate will be the baseline value, and the fixed factor will be the group. A small group analysis will also be performed to explore the differences in the effect of DJS among the peri- and postmenopausal participants.

### Data management and monitoring

Any research-related information collected during the course of the study will be recorded in the clinical report form. Periodic monitoring will be carried out to ensure the quality of the data by qualified, independent monitors. The monitors will verify that the records of the clinical report form are consistent with the original documents and that the practice of the clinical trial conforms to the protocol approved by the institutional review board.

### Compensation

An insurance contract will be concluded to cover compensation to participants who might be injured during the study. In the case of clinically significant damage resulting from adverse drug reactions caused by the medication used in this study, the subject will be financially compensated subject to this insurance contract and the regulations of the clinical trial center.

## Discussion

Since its first description as a herbal prescription for all kinds of female abdominal diseases in an old classical text of ancient Chinese medicine [33], DJS has been widely used in various conditions in gynecology in traditional East Asian medicine. The medication, called Danggwijakyaksan (當歸芍藥散, 당귀작약산), Danger Shaoyao san (当归芍药散), and Toki-shakuyaki-san (当帰芍薬散, トウキシャクヤクサン) in Korea, China, and Japan, respectively, is one of the most commonly used prescriptions for menopausal syndrome in postmenopausal women in these countries. As mentioned, modern scientific studies have shown that DJS may be helpful for women suffering from climacteric symptoms via hormonal control [9–11, 13]. In addition, the major indications for the commercial product of DJS, as approved by the regulatory authorities in Korea and Japan, include climacteric syndrome [10].

However, there is still a lack of rigorously designed randomized controlled trials to assess the effects of DJS on climacteric syndrome in peri- and postmenopausal women, which is why we intend to explore the efficacy and safety of DJS and investigate the feasibility of a clinical study for this issue. The Standard Protocol Items: Recommendations for Interventional Trials (SPIRIT) checklist for this protocol is given in Additional file 1. The findings acquired from this study will inform the design of a confirmative, full-scale randomized controlled trial in the future.

One characteristic of this study is that it is designed based on pattern identification, a theory of diagnosis and a therapeutic system that is unique to traditional East Asian medicine. Danggwijagyaksan has been used predominantly in patients who are identified as having blood-deficiency patterns under this system. Therefore, this study will only include patients who have been identified as having a pattern of blood-deficiency using the Blood Deficiency Scoring System questionnaire in the screening phase. We are working on another pilot study to evaluate the efficacy and safety of Gyejibongnyeonghwan (also called Guizhifulingwan or Keishibukuryogan in China and Japan) for the treatment of climacteric syndrome in peri- and postmenopausal women who are diagnosed with blood stasis patterns under the pattern identification system [12]. A series of these studies involving the unique theory of traditional medicine is expected to provide a basis not only for the efficacy and safety of the herbal medicine for the treatment of climacteric syndrome but also for the means of applying the old and historical concepts of traditional medicine in East Asia in clinical settings today. It is expected that the results of these studies will be able to provide basic data to guide physicians or traditional medical doctors at clinics more clearly in prescribing candidate herbal drugs, such as DJS or Gyejibongnyeonghwan, for patients with climacteric syndrome.

The first limitation of this study is that the primary outcome measure is the Menopause Rating Scale, which is entirely dependent on the participants’ subjective assessments. To compensate for this problem, we will also measure the levels of female hormones, such as follicle-stimulating hormone and estradiol, as objective indicators of changes in the menopausal condition.

The second limitation is the incompleteness of the placebo. The intervention in this study is a granule-form herbal medication acquired from the water extract of a mixture of medicinal plants that has a unique taste and flavor. The appearance of the control intervention, including the color, formulation, and packaging, will be identical to that of DJS, but it is very difficult to reproduce the taste and flavor precisely. To overcome this, we will do our best to ensure that the taste, flavor, and appearance mimic DJS as perfectly as possible. In addition, to ensure that these efforts were successful, we will assess the achievement of participant blinding after the end of the treatment period.

Another limitation is that the scale of this study is too small, and the duration of medication administration and follow-up is short. This is because of the time and financial constraints of the research projects supporting this trial. However, based on the findings that will be acquired from this exploratory pilot study, we will be able to design a further study with improved treatment and follow-up durations.

### Trial status

The recruitment of participants is currently underway.

## Additional file

Additional file 1: SPIRIT 2013 checklist. (DOC 124 kb)

## Abbreviations

DJS: Danggwijagyaksan
SAS®: Strategic Applications Software
SPIRIT: Standard Protocol Items: Recommendations for Interventional Trials
WHOQOL-BREF: World Health Organization Quality of Life-BREF
